# The efficacy of interventions to protect crops from raiding elephants

**DOI:** 10.1007/s13280-021-01587-x

**Published:** 2021-06-25

**Authors:** Robert A. Montgomery, Jamie Raupp, Methodius Mukhwana, Ashley Greenleaf, Tutilo Mudumba, Philip Muruthi

**Affiliations:** 1 Research on the Ecology of Carnivores and Their Prey (RECaP) Laboratory, Department of Fisheries and Wildlife, Michigan State University, East Lansing, MI 48824 USA; 2 Wildlife Conservation Research Unit, Department of Zoology, The Recanati-Kaplan Centre, University of Oxford, Tubney House, Abingdon Road, Tubney, Oxon OX13 5QL UK; 3 African Wildlife Foundation, Uganda Office, Plot 9 Bukoto Crescent, Naguru, P.O. Box 37346, Kampala, Uganda; 4 Department of Zoology, Entomology and Fisheries Sciences, Makerere University, P.O. Box 7062, Kampala, Uganda; 5 African Wildlife Foundation, Headquarters, Ngong Road, Karen, P.O. Box 310, 00502 Nairobi, Kenya

**Keywords:** Coexistence, Conservation, Crop raiding, Elephant, Human-wildlife conflict, Interventions

## Abstract

Both African elephants (*Loxodonta* spp.) and the Asian elephant (*Elephas maximus*) across their range come into conflict with people because of their crop-raiding behavior, which presents profound impediments to farmer livelihoods. In response, a series of interventions, designed to reduce elephant crop raiding have been applied. Based on an extensive review of elephant crop-raiding studies published over a 31-year period, we identified four primary categories of interventions including: (i) detection efforts; (ii) preemptive measures; (iii) fencing and trenches; and (iv) deterrent techniques. The interventions reported to be most effective involved chili peppers (i.e., fences, spray, and briquettes) and crop guarding coupled with deterrents. The extent to which these interventions can be applied more widely is unclear as only two studies examined efficacy across sites in more than one country. Thus, future inquiry should evaluate the ability of effective interventions, or indeed a combination of interventions, to be applied across the range of elephants to reduce crop raiding at scale.

**Supplementary Information:**

The online version contains supplementary material available at 10.1007/s13280-021-01587-x.

## Introduction

In an increasingly urbanized and human-dominated world, wildlife often compete with people for access to land and food resources. The conflict that can grow out of this apparent competition presents threats to human livelihood and the persistence of wildlife populations and habitats (Parker and Graham 1989; Ceballos and Ehrlich 2002; Thirgood et al. 2005; Woodroffe et al. 2005). Conflicts result from human-wildlife interactions that yield negative outcomes for one or both parties (Redpath et al. 2015) and takes many different forms (Macdonald 2016). They can be as seemingly benign as raccoons (*Procyon lotor*) denning in suburban American homes to as severe as hyenas African lions (*Panthera leo*) killing livestock or harming people in sub-Saharan Africa (O’Donnell and DeNicola 2006). Despite this diversity, conflict tends to be most damaging for people and, correspondingly intense for wildlife, when the interactions involve large mammals (Choudhury 2004; Kolowski and Holekamp 2006; Hegel et al. 2009; Abade et al. 2019). Species in the order Carnivora, infra-order Ungulata, and order Proboscidea, for instance, have been disproportionately persecuted by humans in response to real or perceived conflict (Cardillo et al. 2006; Darimont et al. 2009; Chapron et al. 2014; Montgomery et al. 2020a). This persecution, which takes both preemptive and retaliatory forms, is principally motivated by threats to the security of food, property, and human well-being (Decker and Chase 1997; Conover 2001; Treves and Karanth 2003; Redpath et al. 2013). Consequently, human-wildlife conflict is one of the main mechanisms driving population declines of large carnivores and large herbivores around the world (Hoare 2000; Ripple et al. 2014, 2015; Montgomery et al. 2018a).

Across the depth and breadth of human-wildlife conflict research, various applied management actions and research-informed conservation practices have been implemented to promote coexistence among humans and wildlife (Hoare 2000; Nelson 2003). Such efforts have involved assessments of human perceptions, attitudes, and normative behaviors in relation to wildlife, compensation schemes for property loss caused by wildlife, fortification of enclosures promoting food security, and various measures designed to alter animal movement and behavior in relation to human-dominated landscapes (Madden 2004; Hoffmann et al. 2017; Kissui et al. 2019; Meena 2020). However, the efficacy of these interventions are seldom assessed, contributing to evident divides among the research and policy spheres (Kapos et al. 2009; Artelle et al. 2018; Montgomery et al. 2020b). Such ‘knowing-doing’ or ‘research-implementation’ gaps hamper efforts to protect human interests and can simultaneously stymie conservation practice (Knight et al. 2008; Montgomery et al. Montgomery et al. 2018b; Gray et al. 2020). While the research-implementation gap is evidential across disciplines and spatial extents, it is particularly influential when affecting conservation in the Global South (Knight et al. 2008; Sunderland et al. 2009; Gossa et al. 2015). The Global South is comparatively wildlife-rich and also experiences some of the highest human population growth rates on the planet (Gerland et al. 2014; Venter et al. 2016; Crist et al. 2017). These dynamics tend to increase human-wildlife interactions which could intensify conflict (Douglas-Hamilton 1987; Barnes et al. 1991; Woodroffe 2000; Wittemyer et al. 2008). Thus, effective measures that can quantifiably reduce human-wildlife conflict and lead to policy formation designed to alleviate this conflict are urgently needed.

African and Asian elephants (*Loxodonta* spp. and *Elephas maximus*) are the subjects of intense human-wildlife conflict in many parts of the Global South (Osborn and Parker 2003; Fernando et al. 2008; Goswami and Vasudev 2017; Mumby and Plotnik 2018). This conflict is most often triggered by elephant raiding of agricultural crops (O’Connell-Rodwell et al. 2000; Chiyo et al. 2005; Webber et al. 2011). Voracious consumers, elephants can devour multiple hectares of crops in a single night (Naughton 1999; Davies et al. 2011). These incidents can devastate the livelihoods of affected farmers (De Boer and Ntumi 2001; Sitati et al. 2005; Barua 2014). Further, retaliation over real or perceived crop raiding can involve the discriminate and indiscriminate maiming/killing of elephants (Hoare 1995; O’Connell-Rodwell et al. 2000; Nelson 2003; Dunham et al. 2010). Elephants are species of conservation concern globally, and crop raiding presents a major sustainability challenge (Nelson 2003). To address this challenge, we conducted a review to; (i) evaluate the variety of interventions deployed to reduce elephant crop raiding and (ii) explore the relative effectiveness of these interventions to deter elephants. Here, we synthesize the results of this review to identify the techniques that show promise in reducing elephant crop raiding. We examine how these interventions might be applied more widely to maximize potential benefits to human communities that are in conflict with elephants across their range. We discuss the implications of this review for research-informed interventions focused on jointly providing farmer food security and elephant conservation.

## Materials and methods

We conducted a review of both peer-reviewed and gray (i.e., non-peer-reviewed) literature (completed on 31 December, 2019), that evaluated elephant crop raiding. We placed no constraint on the year of publication so as to capture studies published on this topic. Our review of the peer-reviewed literature was executed in the Web of Science search engine with the following terms: (elephant) AND (crop raiding OR crop-raiding OR crop damage). We searched the gray literature in Google using the same terms as above while including a file type as .doc, .docx, or .pdf anticipating that gray literature reports would be available online as either Microsoft Word Files or PDFs. We then assessed all literature returned from the peer-reviewed and gray sources, retainingthose studies that had objectives that were consistent with our analysis. We eliminated studies that did not assess elephant crop raiding, focused purely on elephant ecology, did not test an intervention to reduce elephant crop raiding in a real-world landscape, and those that were reviews or meta analyses. Among the retained studies, we recorded: (i) the research site; country, and continent where the study was located; (ii) the type of intervention implemented to reduce elephant crop raiding; (iii) whether the cost of the application and maintenance of each intervention was provided; and (iv) the reported effectiveness of that specific intervention. Though efficacy can be perceived as a broad term potentially involving a variety of success indicators, we centered our efforts on quantifying the methodologies used to evaluate the efficacy of interventions to deter crop-raiding elephants or reduce crop damage from elephants.

## Results

**Fig. 1:**
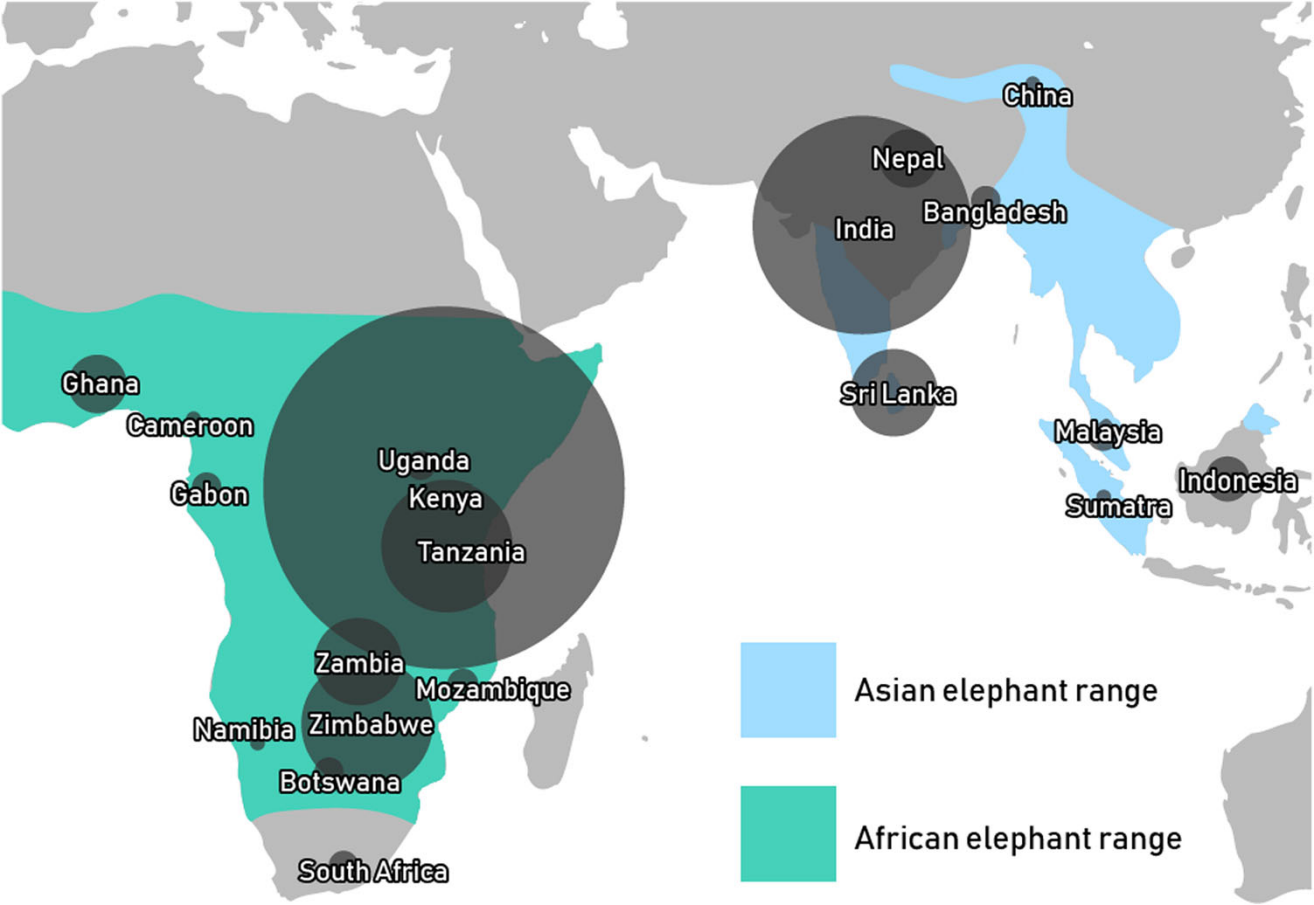
The spatial distribution of research evaluating interventions designed to reduce elephant (*Loxodonta* spp. and *Elephas maximus*) crop-raiding as inferred by a review of 95 studies published between 1993 and 2019

Our literature review returned a total of 280 studies, published between 1988 and 2019, that broadly examined elephant crop raiding, human-elephant conflict, and elephant ecology. Upon examination of each of these studies, we found 185 of them to be inconsistent with our research objectives (i.e., did not directly test interventions designed to reduce elephant crop raiding). Compensation programs were described in some studies, but we did not consider them to be interventions to reduce elephant crop raiding. Rather, they were post-hoc methods that were designed to reduce human retaliation when elephant crop-raiding had already occurred. For these reasons, we did not consider compensation programs within our study of elephant crop-raiding interventions. Among the 95 studies that we retained for further analysis, the majority (82%, *n* = 78) were published in peer-reviewed journals and 17 derived from gray literature sources (For a list of references please see Supplementary Materials, Appendix 1). These 95 studies, with publication dates ranging from 1993 to 2019, were situated among 64 research sites in 20 countries across the range of both African and Asian elephants (Fig. 1). Spatial patterning of these studies were apparent in East Africa (namely Kenya and Tanzania) and South Asia (namely India and Sri Lanka; Fig. 1). Three of these studies (~ 3%) tested interventions in more than one country.

**Fig. 2:**
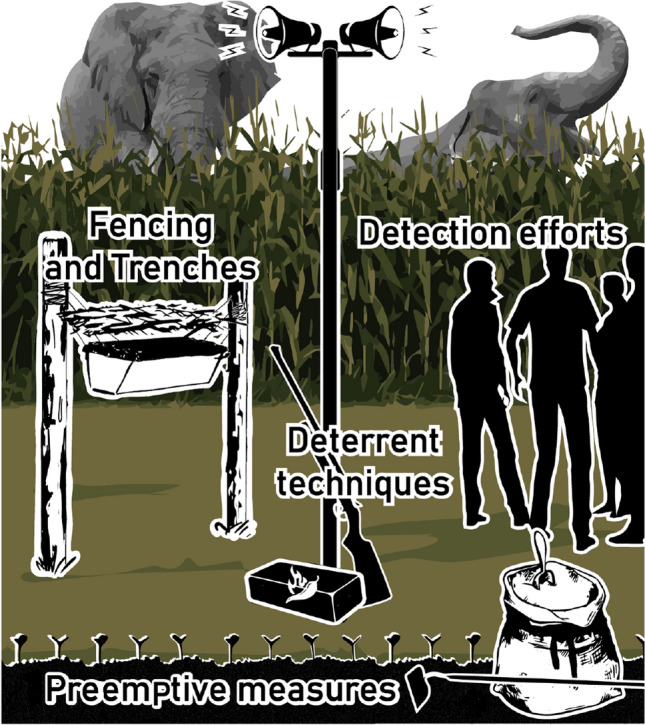
Crop-raiding behavior threatens the livelihood of farmers across the range of African and Asian elephants (*Loxodonta* spp. and *Elephas maximus*). In response, four primary categories of interventions have been tested

We identified four primary categories of interventions designed to reduce elephant crop-raiding among these studies. These included; (i) preemptive measures; (ii) deterrent techniques; (iii) detection efforts; and (iv) fencing and trenches (Fig. 2). It is important to note that we appreciate the distinction between deterrents (i.e., inhibiting animal consumption) and repellents (i.e., altering animal movement trajectories; see Dethier et al. 1960). We use ‘deterrent’ throughout this review given that the interventions herein were designed to reduce elephant crop raiding (i.e., an act of consumption). That being said, we acknowledge that many of these interventions could have served the purpose of both repellant and deterrence. The majority (57%, *n* = 56 of 95) of the studies in our review analyzed technique(s) among one primary intervention category; 17 studies (18%) assessed techniques among two primary intervention categories; 19 studies (20%) evaluated techniques across three intervention categories; and five studies (5%) analyzed techniques among all four primary intervention categories.

**Fig. 3:**
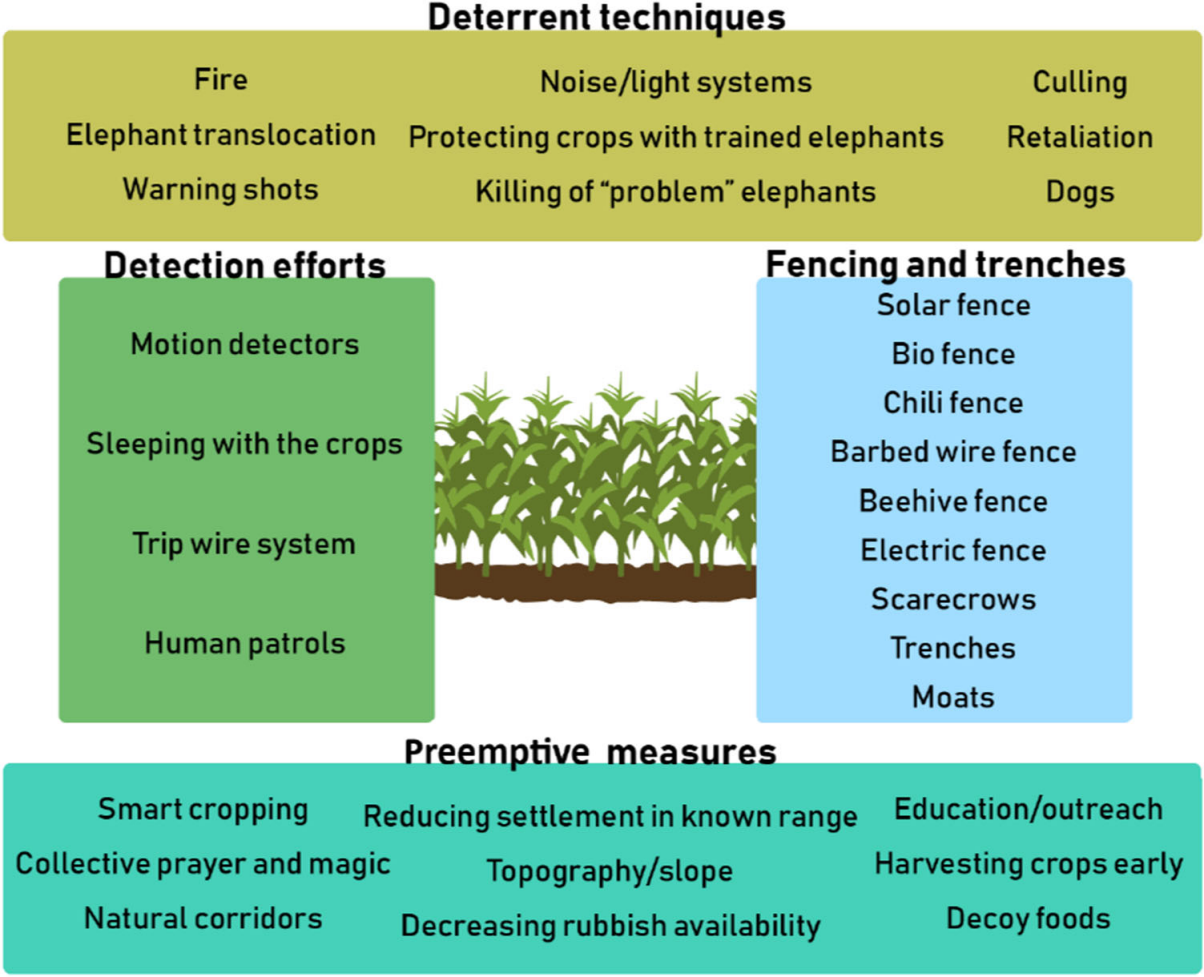
The four primary categories of interventions, and the corresponding examples of the variety of actions within each category, designed to reduce elephant (*Loxodonta* spp. and *Elephas maximus*) crop-raiding. These categories and actions were detected among an extensive review of 95 studies published between 1993 and 2019

In total, there were 31 different intervention techniques tested among this literature (Fig. 3). The most common interventions tested were within the fencing and trenches category (62%, *n* = 59 of 95 studies) followed by deterrent techniques (51%, *n* = 48 of 95 studies),detection efforts (41%, *n* = 39 of 95 studies), and preemptive measures (20%, *n* = 19 of 95 studies). The fencing and trenches category included several fencing options (i.e., solar, bio, chili, barbed wire, and beehive) that might be implemented separate to, or in tandem with, trenches and moats (Fig. 3). Motion-activated and tripwire systems along with human patrolling or sleeping with crops were among the techniques featured in the detection efforts category (Fig. 3). Preemptive measures included the strategic placement of crops in areas of low likelihood of elephant occurrence, decreasing rubbish, providing education and outreach efforts relating to best practices, and planting unpalatable decoy foods (Fig. 3). Elephant translocation, culling, and retaliation were among the techniques deployed in the deterrent category (Fig. 3).

The effectiveness of these intervention techniques at deterring crop-raiding elephants and reducing crop damage was evaluated among 83% (*n* = 79 of 95) of the studies. Assessments of efficacy included quasi-experimental designs (i.e., case-control), direct observation, post-hoc analyses of elephant movement paths (via telemetry technology), and semi-structured surveys gauging human perceptions. Accordingly, estimates of efficacy were highly variable involving: (i) percent reductions in crop raiding among comparisons of test and control plots; (ii) proportion of respondents that believed a technique to be effective; and (iii) deviation in movement paths of collared elephants, among others. No single technique was deemed to be 100% effective at deterring crop-raiding elephants. However, several techniques were cited as being most effective at reducing elephant crop-raiding (Table 1). Chili pepper approaches, including fences, spray, and briquettes, were cited as being effective among 28% (*n* = 22 of 79) of the studies across eight countries. Crop guarding was cited among 23% of the studies in 11 countries followed by electric fences at 18% across seven countries. All other techniques that were deemed to be effective occurred in ≤10% of all studies. Interventions that were cited as being ineffective including metal and electric fencing (*n* = 4), beehive fences (*n* = 1), and elephant translocations (*n* = 1). Among these studies, 18 (19%) provided estimates of the costs of implementation of these interventions. The costs were highly variable across studies, ranging from $22 for a beehive to > $1,000,000 for metal fencing of the periphery of a conservancy (Table 2).

**Table 1:** The intervention techniques that were cited to be effective at reducing elephant (*Loxodonta* spp. and *Elephas maximus*) crop raiding, as determined by quasi-experimental, direct observation, human perception surveys, and elephant movement path designs

Intervention technique	Count	Proportion	Number of countries	References^m^
Chili pepper approaches	22	0.28	8^a^	1–22
Fences (16)				
Spray (4)				
Briquettes (2)				
Crop guarding	18	0.23	11^b^	23–40
Electric fences	14	0.18	7^c^	41–54
Active defense (noise, projectiles, shots, fire)	8	0.10	6^d^	55–62
Beehive fences	7	0.09	5^e^	63–70
Trenches	4	0.05	2^f^	71–74
Detection systems	4	0.05	2 ^g^	75–78
Smart cropping	3	0.04	3 ^h^	79–81
Playbacks	2	0.03	2^i^	82–83
Decreasing rubbish availability	1	0.01	1^j^	84
Spotlights	1	0.01	1^k^	85
Natural corridors	1	0.01	1^l^	86

These techniques were reported among 79 of 95 studies that tested the efficacy of elephant crop-raiding interventions. As numerous techniques could have been tested among any given study, the count column exceeds 79 and, correspondingly, the proportion totals >1.00

^a^Botswana, Ghana, India, Kenya, Mozambique, Tanzania, Zambia, Zimbabwe; ^b^Bangaladesh, India, Indonesia, Kenya, Nepal, Sri Lanka, Sumatra, Tanzania, Uganda, Zambia, Zimbabwe; ^c^India, Indonesia, Kenya, Malaysia, Namibia, Zambia, Uganda; ^d^Ghana, India, Kenya, Nepal, Sri Lanka, Zimbabwe; ^e^Gabon, India, Kenya, Mozambique, Tanzania; ^f^India, Indonesia; ^g^Kenya, South Africa; ^h^Ghana, Nepal, Zambia; ^i^India, Sri Lanka; ^j^Zimbabwe; ^k^India; ^l^Tanzania; ^m^For a list of references please see Supplementary Materials, Appendix S2

**Table 2:** Descriptions of the costs of intervention techniques designed to reduce elephant (*Loxodonta* spp. and *Elephas maximus*) crop raiding

References	Intervention	Description
Hahn et al. (2017)	Aerial drone	“The cost of five teams responsible for 607 km^2 in Tarangire–Manyara was estimated to be USD $15 520 for 1 year, and all drones remained operational for the duration of the study.” p. 1
King et al. (2009)	Beehive fence	“Costs for the beehive fence based on using traditional log beehives were approximately US$315 per 100 m.” p. 134
Scheijen et al. (2019)	Beehive fence	“The cost of constructing the fence was TZS 9031 (USD 4.25/m).” p. 94
King et al. (2011)	Beehive fence	“149 beehives were constructed on site and deployed between June and August 2008 and the remaining 21 in April 2009 at a cost of US$22 per hive. This resulted in 1700 m of beehive fences incorporating 170 beehives, around the boundaries of seventeen community farms.” p. 434
Branco et al. (2019)	Beehive fence and chili pepper fence	“Construction of a beehive fence with 15 hives in our study cost $773 USD in materials. The hives themselves comprised the majority of the cost ($33.50 USD apiece for Kenyan top bar hives), with other equipment and supplies (bee attractant, hardwood poles, yellow paint, bailing twine, nails, wire, bee brush, and gloves) totaling $270 USD.” p. 6
Zimmermann et al. (2009)	Chili briquette, spotlight, and electric fence	“We have assisted communities with the installation of simple electric fences at three sites… Although this is our most expensive intervention option (at a cost of approx £1400 / Rs1 11 000 per kilometre).” p. 37
Baishya et al. (2012)	Chili pepper fence	“The cost of material for putting up the chilli fence for the test plot was nearly Rs. 20 000 (US$ 400).” p. 12
Chang’a et al. (2016)	Chili pepper fence	“The cost of the materials for fencing a hectare of crops was approximately $14 in 2015 ($35 per acre), though recurring costs can be reduced by recycling fence poles, cloths, and ropes over several harvest seasons.” p. 924
Kiiru et al. (2006)	Chili pepper fence, trip wire with air horn, and fireworks	“The price for an assortment of fireworks ranged from Kshs 30 (USD 0.40) to 100 (USD 1.33), well within financial reach of farmers in the Amboseli region.” p. 2 Approx. cost for the first few pilot sound devices were Kshs 4 000v(USD 55), with the air release/handle as the most expensive part.” p. 3 “While it is easy to set up trip wires, and cost for the materials is relatively low, about USD 32 per acre (280 m strip), they are prone to community management deficiencies.” p. 4 “The cost of constructing a watchtower ranged from as low as Kshs 200 (USD 3) if the farmers were able acquire poles on their land, to about Kshs 1500 (USD 20) if they had to purchase them.” p. 5 “Approx. cost a of chili-tobacco rope per acre (280 m) crop field: $32.” p. 6
Osborn and Parker (2002)	Chili pepper spray	“The capsium spray that was tested is relatively expensive ($5 per discharge) and was imported from the United States” p. 677
Le Bel et al. (2010)	Chili pepper gun	“For the present study, the costs of both dispensers as handmade prototypes were less than USD 50. Taking into account the cost of ping-pong balls (USD 0.11 per ball), commercial aerosol as a propellant (10 cc spray costing USD 0.13) and imported chilli oil extract rating 250.000 SHU (30 cc per ball costing USD 0.96), the cost of repelling an elephant is estimated at USD 1.20.” p. 86
De Boer and Ntumi (2001)	Electric fence	“The construction and maintenance of the 38-km electric fence is estimated at US$41 100 per year.” p. 57
Thouless and Sakwa (1995)	Electric fence	“Construction costs for this fence (electric) were approximately $2500/km and annual maintenance. A stone was built along the southern boundary with flat stones, at a cost of approximately $3500/km. A 6 km six strand fence (electric) ran along the eastern boundary, construction costs were approximately $4000/km.” p. 103
Smith and Kasiki (2000)	Electric fences	“In 1996 an electric fence was built to reduce HEC in Taita Taveta at an estimated cost of US $324 000.” p. vii “In 1995/96 an electric fence was constructed between Ndara and Ndi as an additional HEC mitigation measure… It’s estimated installation cost was US$10 800 per km and the calculated annual cost of maintenance per km is US$1100.” pp. 23–24
Kioko et al. (2008)	Electric fences	“The Kimana and Namelok fences were completed in 2000 at a cost of US$9000/km with financial support from European Union.” p. 53
O'Connell-Rodwell et al. (2000)	Electric fence and trip wire	“The electric fence at Lianshulu cost approximately US$5900…Trip-alarms were relatively inexpensive (US$78).” p. 387
Gross et al. (2017)	Smart cropping	Table 4 provides production costs (USD/kg) for various medicinal and aromatic crops. p. 34

These costs were reported among 18 of the 95 studies that tested the efficacy of elephant crop-raiding interventions

## Discussion

The crop-raiding behavior of elephants jeopardizes the security of affected farmers and endangers elephant conservation. Thus, elephant crop-raiding fueling human-elephant conflict represents a long-term intractable problem without clear-cut solutions (Nelson 2003; Dublin and Hoare 2004). We identified 31 interventions designed to reduce elephant crop raiding that were tested among 64 research sites distributed in 20 countries across the range of elephants, mostly in East Africa and Southern Asia (Fig. 1). This spatial pattern may result from the presence of comparatively large populations of elephants with comparatively dense human populations and thus, human-wildlife interactions could plausibly be higher (Blanc 2008; Choudhury et al. 2008; Chase et al. 2016; Thouless et al. 2016). This patttern could also simply reflect where research effort tends to be located. Nevertheless, the fact that these spatial patterns persist exemplifies the critical importance of resolving conflict triggered by elephant crop raiding.

Human retaliation to elephant crop raiding and other motivations of illegal killing can have negative population-level consequences on elephant demography (Hoare 2000; Kahindi et al. 2010; Burn et al. 2011). There are now three species of elephants that are recognized by the International Union for Conservation of Nature (IUCN) with Asian elephant (*Elaphus maximus*) and African savanna elephant (*L. africana*) listed as endangered and African forest elephants (*L. cyclotis*) determined to be critically endangered (Williams et al. 2020; Gobush et al. 2021a, b). Thus conservation action for these species is urgent, with human-elephant conflict as a major issue to be addressed. Here, we have shown the range of solutions that are being applied to the elephant crop-raiding problem across the elephant ranges. Two of the studies that we examined, evaluated interventions across >1 country. One assessed elephant crop raiding in the borderlands between Kenya and Tanzania (Osipova et al. 2018). Despite the administrative boundaries between these countires, the borderland region represents one large ecosystem. On the other hand, Gross et al. (2019) selected two African sites and two Asian sites to test a series of elephant crop raiding interventions providing a template for robust comparison of elephant crop-raiding interventions across scales. Nevertheless, the extent to which interventions to reduce elephant crop-raiding are generalizable remains unclear. Instead, the considerable socio-ecological variation across the range of African and Asian elephants suggests that effective management will need to draw upon the full range of possible responses. For instance, the sustainability of elephant crop-raiding interventions directly depends upon the willingness of affected farmers and communities to uptake these techniques, and this is likely to be site-specific. One important innovative is the human heritage-centered conservation (HHCC) framework that highlights the importance of not presuming that a conservation solution vetted in one site should necessarily be applicable to another (see Montgomery et al. 2020b). To do so would be to propagate the myths of *whiz*-*bang solutions* (Montgomery et al. 2020b). Instead, we recommend that additional research be focused on the application and testing of interventions designed to reduce elephant crop raiding among numerous sites across the range of African and Asian elephants. The costs associated with developing and applying intervention techniques will be a critical factor, but we found that cost estimates were provided in < 20% of the studies (Table 2). Thus, accurate reporting of the costs inherent to conservation solutions remains an area of important need for policy makers (Muruthi 2005; Karidozo and Osborn 2015). Without such information, the applicability of interventions more widely is limited, representing an avenue of future, and progressive, research-informed conservation work.

We also found that the methods of measuring the effectiveness of interventions designed to reduce elephant crop-raiding were highly variable across studies. While 83% (*n* = 79 of 95) of the studies that we reviewed reported some measure of efficacy, techniques to do so included human perception surveys, quasi-experimental designs, and examinations of elephant movement paths from telemetry, among others. The high degree of variability limited the comparability of these interventions across sites. For instance, it is challenging to compare an intervention deemed to be effective via semi-structured surveys to that which was found to be effective when comparing test and control sites. Thus, we support the call of Denninger Snyder and Rentsch (2020) to embed more quantitiative rigor in the assessment of interventions designed to reduce negative human-elephant interactions. They provided a conceptual framework where effectiveness was predicted as a function of the efficacy of the intervention along with the ability of that intervention to be maintained (i.e., cost, feasibility, and resources) by affected farmers over time. These are the types of changes that are needed to properly evaluate whether certain intervention techniques can be sustained and scaled across sites.

Despite this context, the intervention technique most commonly cited as being effective involved chili pepper approaches. As olfaction is a key sense used by elephants when foraging (Plotnik and de Waal 2014; Schmitt et al. 2018) it is believed that the fragrance of chili peppers (*Capsicum*) can act as both a repellent and deterrent (Le Bel et al. 2015; Karidozo and Osborn 2015). Chili peppers have been widely used (i.e., validated for efficacy across eight countries), and in multiple forms (Table 1). These included chili peppers in the form of: (i) plants; (ii) grease on fence lines; (iii) spray; and (iv) briquettes. The spatial configuration of these techniques were described as being strategic so as to cover the extent of farmers’ crops. For instance, farmers might plant chili peppers as a buffer around their farm, regularly place chili pepper grease on string or metal fences around their property, apply chili pepper spray before, during, or after elephant interaction, or burn chili pepper briquettes regularly, or semi-regularly. However, emerging evidence suggests that the application of chili peppers as an intervention must consider community-level scales given the potential to essentially push elephants from one farmer’s property onto another (Le Bel et al. 2015). These interventions, like many others that relate to elephant crop raiding, can magnify the threat for neighboring farmers that have not employed, or are not protected by, the interventions. Furthermore, methods such as grease, spray, and briquettes typically offer short term impacts with the magnitudes of effect varying as a function of prevailing weather conditions (Hedges and Gunaryadi 2010; Chelliah 2010; Pozo et al. 2019). The chili pepper plants, on the other hand, can provide year-round impacts providing that fruit is regularly being produced (Chang’a et al. 2016). And once produced, chili pepper fruit can be sold in whole or dried form creating a secondary cash crop for farmers (Parker and Osborn 2006; Hedges and Gunaryadi 2010).

The next most commonly cited effective intervention technique was crop guarding. Crop guarding, particularly when coupled with broader community vigilance activities, involves people sleeping among the crops so as to rapidly detect advancing elephants (Shaffer et al. 2019). However, in the absence of additional interventions, crop guarding can do little more than provide a real-time alert system. Thus, this technique was often coupled with defense mechanisms including humans shouting, dogs barking, projectiles (i.e., sticks and stones), warning shots, and fire (Musyoki 2014). Crop guarding is also a highly risky intervention, especially at night when cooperation with fellow farmers and wildlife authorities become less accessible (Osborn and Parker 2003; Graham et al. 2012). It is not uncommon for people to be trampled and killed by elephants in these settings. In 2007, for example, 50 people, many of them farmers, were estimated to be killed by elephants in Sri Lanka alone (Santiapillai et al. 2010). Gaining further insights into the relationship between human deaths and crop guarding across the range of African and Asian elephants requires more thorough record keeping on farmer mortality (Bandara 2002). Undoubtedly, crop guarding offers a rudimentary detection system with people positioned in lookouts. More advanced forms of technology, including autonomous elephant-detected systems should be pursued to replace the need for farmers and fellow community members to sleep among their crops.

The efficacy of both electric fencing and beehive fences depends on continued maintenance that was acknowledged to be both time-consuming and costly (Thouless and Sakwa 1995; Kioko et al. 2008; Noga et al. 2015). Estimates of the annual cost of maintaining electric fencing in Kenya, for instance, were found to exceed the costs of elephant crop raiding (Evans 2015). However, even when properly implemented, a comparative study found no statistical difference in human-elephant conflict in the six-month period before and after the implementation of electric fencing (Smith and Kasiki 2000). Beehives are notoriously difficult to maintain, both in terms of the structure of the hives themselves as well as the process of keeping the bees in residence (Tesfaye et al. 2017; Gratzer et al. 2019). These hives are often affixed to fences, poles, or trees distributed along the periphery of farmers’ crops, assuming that elephants are deterred by the cues of bees (King et al. 2009, 2011). However, such hives can be pushed over or broken by advancing elephants and may only be effective if the hive has high levels of bee activity (Vollrath and Douglas-Hamilton 2002; Ngama 2016). For these reasons, the effects of beehive fences may be short term (Nair and Jayson 2016) or perhaps even ineffective (Ndlovu et al. 2016). That being said, beehive fences have been found to deter elephants in study sites where there is sustained engagement from non-governmental organizations such as in Laikipia County, Kenya (see King et al. 2009, 2011). Given the complex technology that is bee-keeping, reliable and long-term collaboration with farmers may be needed to ensure that this can be an enduring solution against elephant crop raiding.

Another of the intervention techniques cited as being ineffective was elephant translocation with so-called ‘problem’ elephants, those that were deemed to regularly raid crops, being tranquilized, moved, and released into new locations (Dublin 2003). Fernando et al. (2012) monitored 16 of these elephant translocations in Sri Lanka and found that human-elephant conflict actually increased via the processes of the translocated elephants moving widely to return to their home site, as part of exploratory behaviors, or when establishing home ranges in new areas. Not only that, but translocation has been found to be one of the ways of disrupting the integrity of elephant herd structures, increasing stress in individuals animals and leading to intense forms of downstream human-elephant conflict (Bradshaw et al. 2005). Any elephant crop-raiding intervention that actually yields a result opposite to that intended must be critically scrutinized. It is important to note that elephant crop raiding tends to be more intense nearer to protected areas or known elephant migration routes (Gubbi 2012; Hoffmeier-Karimi and Schulte 2015). Thus, farms that are adjacent to these areas are subject to higher levels of crop-raiding risk and should be considered priority locations for the prescriptions of crop-raiding interventions.

In summary, while many of the interventions that we reviewed were effective to some degree, we believe that future research on human-elephant conflict should focus, not only on these techniques, but also on the rigorous application and development of new technologies capable of reducing elephant crop-raiding. For instance, automated systems that can autonomously detect elephants and alert farmers (via SMS communication, for instance) are now emerging (Zeppelzauer et al. 2015; Ramesh et al. 2017). These systems can issue built-in responses (including lights and noises) to deter elephants once detected (Asimopoulos 2016). Such systems can also be randomized so as to decrease the probability of elephants becoming habituated to the responses (Shaffer et al. 2019). Nevertheless, maintaining such equipment can become a limiting factor for alert systems as a deterrent, so cost effective recharging stations should be installed (O’Connell-Rodwell et al. 2000). Playbacks, such as human shouting, sympatric predator growls, or even elephant vocalizations, have shown promise in this respect (Thuppil and Coss 2013; 2016). Additional techniques, even those that are not yet fabricated, will be needed. We support efforts for renewed innovation to rigorously develop new technologies that can be productively applied and validated widely to help farmers affected by elephant crop raiding. To create a more resilient future for elephants and people, it will be paramount to learn from the interventions featured in this review while simultaneously working to engineer the next generation of interventions. As human settlements and elephant habitat become increasingly interconnected, the frequency and severity of conflict will likely rise (sensu Harich et al. 2013; Redpath et al. 2013). Thus, it is imperative that novel and original interventions be rapidly deployed to protect the physical, mental, and economic health of people that share their land with elephants and to promote the conservation of elephants across their range.

## Supplementary Information

Below is the link to the electronic supplementary material.

Supplementary material 1 (PDF 631 kb)
