# Supplementary material for: The efficacy of interventions to protect crops from raiding elephants
Source: Ambio. 2021 Jun 25;51(3):716–27. doi: 10.1007/s13280-021-01587-x (PMC8800974; doi:10.1007/s13280-021-01587-x)
Supplement: Supplementary file 1 — Supplementary material 1 (PDF 631 kb) [file 13280_2021_1587_MOESM1_ESM.pdf]

Supplementary Materials for

The Efficacy of Interventions to Protect Crops from Raiding Elephants

Appendix 1: Literature review

The 95 studies published between 1993 and 2019 that met our criteria for inclusion in this analysis of the efficacy of interventions designed to reduce elephant crop raiding.

Adjewodah, P., Murphy, A. and Mason, J., 2003. Mitigating elephant crop-raiding: the Red Volta valley experience, Ghana. IUCN report, https://www.iucn.org/sites/dev/files/import/downloads/hecrvvrep.pdf

Adjewodah, P., Oduro, W. and Asase, A., 2012. Functional relationship between crop raiding by the savannah elephant and habitat variables of the Red Volta Valley in north-eastern Ghana. *Pachyderm*, 52 pp. 23-35.

Asimopoulos, S., 2016. *Human-wildlife conflict mitigation in Peninsular Malaysia: lessons learnt, current views and future directions*. Second cycle, A2E. Uppsala: SLU, Dept. of Urban and Rural Development

Baishya, H.K., Dey, S., Sarmah, A., Sharma, A., Gogoi, S., Aziz, T., Ghose, D. and Williams, A.C., 2012. Use of chilli fences to deter Asian elephants-a pilot study. *Gajah*, 36, pp.11-13.

Bandara, R. and Tisdell, Cl., 2002. Asian Elephants as Agricultural Pests: Damages, Economics of Control and Compensation in Sri Lanka. *Natural Resources Journal*. 42.

Baskaran, N. 1995. Crop raiding by Asian elephant in Nilgiri Biosphere Reserve, South India. In: A week with Elephants: Proceedings of the International Seminar on the Conservation of the Asian Elephant (June 1993) (Eds J. C. Daniel and H. S. Datye) Oxford University Press, Bombay.

Branco, P.S., Merkle, J.A., Pringle, R.M., King, L., Tindall, T., Stalmans, M., Long, R.A., 2019. An experimental test of community-based strategies for mitigating human–wildlife conflict around protected areas. *Conservation Letters*, 13(1), e12679.

Chang'a, A., Souza de, N., Muya, J., Keyyu, J., Mwakatobe, A., Malugu, L., Ndossi, H.P., Konuche, J., Omondi, R., Mpinge, A. and Hahn, N., 2016. Scaling-up the use of chili fences for reducing human-elephant conflict across landscapes in Tanzania. *Tropical Conservation Science*, 9(2), pp.921-930.

Chelliah, K., Kannan, G., Kundu, S., Abilash, N., Madhusudan, A., Baskaran, N. and Sukumar, R., 2010. Testing the efficacy of a chilli–tobacco rope fence as a deterrent against crop-raiding elephants. *Current Science*, pp.1239-1243.

Dakwa, K.B., Monney, K.A. and Attuquayefio, D., 2016. Raid range selection by elephants around Kakum Conservation Area: Implications for the identification of suitable mitigating measures. *International Journal of Biodiversity and Conservation*, 8(2), pp.21-31.

Davies, T.E., Wilson, S., Hazarika, N., Chakrabarty, J., Das, D., Hodgson, D.J. and Zimmermann, A., 2011. Effectiveness of intervention methods against crop-raiding elephants. *Conservation Letters*, 4(5), pp.346-354.

De Boer, F., Ntumi, C.P., 2001. Elephant crop damage and electric fence construction in the Maputo Elephant Reserve, Mozambique. *Pachyderm* 30, 57–64.

Evans, L.A., 2015. Fencing the front line: the separation of elephants and cultivation with electrified fences. In fulfillment of the partial requirements for the Doctor of Philosophy degree at the University of Cambridge.

Evans, L.A., Adams, W.M., 2016. Fencing elephants: The hidden politics of wildlife fencing in Laikipia, Kenya. *Land Use Policy* 51, pp. 215–228.

Fazil, M., Firdhous, M., 2018. *IoT-Enabled Smart Elephant Detection System for Combating Human Elephant Conflict*, in: 2018 3rd International Conference on Information Technology Research, ICITR 2018. Institute of Electrical and Electronics Engineers Inc.

Fernando, P., Leimgruber, P., Prasad, T. and Pastorini, J., 2012. Problem-elephant translocation: translocating the problem and the elephant?. *PloS One*, 7(12).

Govind, S.K. and Jayson, E.A., 2013. Efficiency of chilli powder (Capsicum sp.) to deter wild elephants from the crop fields in a tropical area. Millennium Zoology, St. Aloysius College, 14, pp.21-24.

Graham, M.D. and Ochieng, T., 2008. Uptake and performance of farm-based measures for reducing crop raiding by elephants Loxodonta africana among smallholder farms in Laikipia District, Kenya. *Oryx*, 42(1), pp.76-82.

Graham, M.D., Adams, W.M. and Kahiro, G.N., 2012. Mobile phone communication in effective human elephant–conflict management in Laikipia County, Kenya. *Oryx*, 46(1), pp.137-144.

Graham, M.D., Notter, B., Adams, W.M., Lee, P.C. and Ochieng, T.N., 2010. Patterns of crop-raiding by elephants, Loxodonta africana, in Laikipia, Kenya, and the management of human–elephant conflict. *Systematics and Biodiversity*, 8(4), pp.435-445.

Gross, E.M., Drouet-Hoguet, N., Subedi, N., Gross, J., 2017. The potential of medicinal and aromatic plants (MAPs) to reduce crop damages by Asian Elephants (*Elephas maximus*). *Crop Protection* 100, 29–37.

Gross, E.M., Lahkar, B.P., Subedi, N., Nyirenda, V.R., Lichtenfeld, L.L., Jakoby, O., 2019. Does traditional and advanced guarding reduce crop losses due to wildlife? A comparative analysis from Africa and Asia. *Journal of Nature Conservation*, 50, 125712.

Gross, E.M., McRobb, R. and Gross, J., 2016. Cultivating alternative crops reduces crop losses due to African elephants. *Journal of Pest Science*, 89(2), pp.497-506.

Gubbi, S., 2012. Patterns and correlates of human–elephant conflict around a south Indian reserve. *Biological Conservation*, 148(1), pp.88-95.

Guerbois, C., Chapanda, E. and Fritz, H., 2012. Combining multi-scale socio-ecological approaches to understand the susceptibility of subsistence farmers to elephant crop raiding on the edge of a protected area. *Journal of Applied Ecology*, 49(5), pp.1149-1158.

Gunaryadi, D. and Sugiyo, S.H., 2017. Community-based human–elephant conflict mitigation: The value of an evidence-based approach in promoting the uptake of effective methods. *PloS One*, 12(5).

Hahn, N., Mwakatobe, A., Konuche, J., de Souza, N., Keyyu, J., Goss, M., Chang'a, A., Palminteri, S., Dinerstein, E. and Olson, D., 2017. Unmanned aerial vehicles mitigate human–elephant conflict on the borders of Tanzanian Parks: a case study. *Oryx*, 51(3), pp.513-516.

Hariohay, K.M., Munuo, W.A., Røskaft, E., 2019. Human–elephant interactions in areas surrounding the Rungwa, Kizigo, and Muhesi Game Reserves, central Tanzania. *Oryx* 1–9.

Haturusinghe, H.S. and Weerakoon, D.K., 2012. Crop raiding behaviour of elephants in the Northwestern region of Sri Lanka. *Gajah*, p.26.

Hedges, S. and Gunaryadi, D., 2010. Reducing human–elephant conflict: do chillies help deter elephants from entering crop fields?. *Oryx*, 44(1), pp.139-146.

Hoffmeier-Karimi, R.R. and Schulte, B.A., 2015. Assessing perceived and documented crop damage in a Tanzanian village impacted by human-elephant conflict (HEC). *Pachyderm*, 56, pp.51-60.

Hsiao, S.S., Ross, C., Hill, C.M. and Wallace, G.E., 2013. Crop-raiding deterrents around Budongo Forest Reserve: an evaluation through farmer actions and perceptions. *Oryx*, 47(4), pp.569-577.

Jasmine, B., Ghose, D. and Das, S.K., 2015. An attitude assessment of human-elephant conflict in a critical wildlife corridor within the Terai Arc Landscape, India. *Journal of Threatened Taxa*, 7(2), pp.6843-4852.

Jayson, E.A. and Christopher, G., 2008. Human-elephant Conflict in the Southern Western Ghats: a Case Study from the Peppara Wildlife Sanctuary, Kerala, India. *Indian Forester*, 134(10), pp.1309-1325.

Karidozo, M., Ferrel, V.O. 2015. Community Based Conflict Mitigation Trials: Results of Field Tests of Chilli as an Elephant Deterrent. *Journal of Biodiversity and Endangered Species* 3.

Kiiru, W., J. Kioko., and P. Granli. 2006. *Mitigating human-elephant conflict in the Amboseli ecosystem, Kenya. Summary testing of deterrents Year 1*. AERP HEC Project report to US Fish and Wildlife Service.

Kikoti, A.P., Griffin, C.R., Pamphil, L. 2010. Elephant use and conflict leads to Tanzania first wildlife conservation corridor *Pachyderm*, 48:57-66

King, L.E., Douglas-Hamilton, I. and Vollrath, F., 2011. Beehive fences as effective deterrents for crop-raiding elephants: field trials in northern Kenya. *African Journal of Ecology,* 49:431-439.

King, L.E., Lala, F., Nzumu, H., Mwambingu, E. and Douglas-Hamilton, I., 2017. Beehive fences as a multidimensional conflict-mitigation tool for farmers coexisting with elephants. *Conservation Biology*, 31:743-752.

King, L.E., Lawrence, A., Douglas-Hamilton, I. and Vollrath, F., 2009. Beehive fence deters crop-raiding elephants. *African Journal of Ecology*, 47:131-137.

Kioko, J., Kiringe, J. and Omondi, P., 2006. Human-elephant conflict outlook in the Tsavo-Amboseli ecosystem, Kenya. *Pachyderm*, 41:53-60.

Kioko, J., Muruthi, P., Omondi, P. and Chiyo, P.I., 2008. The performance of electric fences as elephant barriers in Amboseli, Kenya. *African Journal of Wildlife Research*, 38:52-58.

Le Bel, S., La Grange, M. and Drouet, N., 2015. Repelling elephants with a chilli pepper gas dispenser: field tests and practical use in Mozambique, Zambia and Zimbabwe from 2009 to 2013. *Pachyderm*, 56:87-96.

Le Bel, S., Taylor, R., Lagrange, M., Ndoro, O., Barra, M. and Madzikanda, H., 2010. An easy-to-use capsicum delivery system for crop-raiding elephants in Zimbabwe: preliminary results of a field test in Hwange National Park. *Pachyderm*, 47: 80-89.

Litoroh, M., Kock, R. and Jachmann, H. 2002. *Evaluation to investigate the feasibility of a proposed translocation of elephants from Arly National Park in Burkina Faso to*

Niokolo-Koba National Park in Senegal. Unpublished Report to IUCN Senegal National Office.

Musyoki, C., 2014. Crop defense and coping strategies: Wildlife raids in Mahiga'B'village in Nyeri District, Kenya. *African Study Monographs,* 35: 19-40

Nair, R.P. and Jayson, E.A., 2016. Effectiveness of beehive fences to deter crop raiding elephants in Kerala, India. *International Journal of Natural and Applied Sciences*, 3, pp.14-19.

Nath, N.K., Lahkar, B.P., Brahma, N., Dey, S., Das, J.P., Sarma, P.K. and Talukdar, B.K., 2009. An assessment of human-elephant conflict in Manas National Park, Assam, India. *Journal of Threatened Taxa*, pp.309-316.

Ndlovu, M., Devereux, E., Chieffe, M., Asklof, K. and Russo, A., 2016. Responses of African elephants towards a bee threat: Its application in mitigating human-elephant conflict. *South African Journal of Science*, 112(1-2), pp.01-05.

Neupane, D., Johnson, R.L. and Risch, T.S., 2017. How do land-use practices affect human—elephant conflict in nepal?. *Wildlife Biology*, 2017(4).

Ngama, S., Bindelle, J., Poulsen, J.R., Hornick, J.L., Linden, A., Korte, L., Doucet, J.L. and Vermeulen, C., 2019. Do topography and fruit presence influence occurrence and intensity of crop-raiding by forest elephants (Loxodonta africana cyclotis)?. *PloS One*, 14(3).

Ngama, S., Korte, L., Bindelle, J., Vermeulen, C. and Poulsen, J.R., 2016. How bees deter elephants: beehive trials with forest elephants (Loxodonta africana cyclotis) in Gabon. *PLoS One*, 11(5).

Njumbi, S., Waithaka, J., Gachago, S., Sakwa, J., Mwathe, K., Mungai, P., Mulama, M., Mutinda, H., Omondi, P. and Litoroh, M., 1996. Translocation of elephants: the Kenyan experience. *Pachyderm*, 22, pp.61-65.

Noga, S.R., Kolawole, O.D., Thakadu, O. and Masunga, G., 2015. Small farmers' adoption behaviour: uptake of elephant crop-raiding deterrent innovations in the Okavango Delta, Botswana. *African Journal of Science, Technology, Innovation and Development*, 7(6), pp.408-419.

Nyhus, P.J. and Tilson, R., 2000. Crop-raiding elephants and conservation implications at Way Kambas National Park, Sumatra, Indonesia. *Oryx*, 34(4), pp.262-274.

Nyirenda, V.R., Chansa, W.C., Myburgh, W.J. and Reilly, B.K., 2011. Wildlife crop depredation in the Luangwa Valley, eastern Zambia. *Journal of Ecology and the Natural Environment,* 3(15), pp.481-491.

Nyirenda, V.R., Myburgh, W.J. and Reilly, B.K., 2012. Predicting environmental factors influencing crop raiding by African elephants (Loxodonta africana) in the

Luangwa Valley, eastern Zambia. *African Journal of Environmental Science and Technology*, 6(10), pp.391-400.

Nyumba, T., 2008. *Coping with Human-Elephant Conflict in Laikipia District, Kenya*. In fulfillment of the partial requirements for the award of the Master of Philosophy degree in Environment, Society and Development of the University of Cambridge.

O'Connell-Rodwell, C.E., Rodwell, T., Rice, M. and Hart, L.A., 2000. Living with the modern conservation paradigm: can agricultural communities co-exist with elephants? A five-year case study in East Caprivi, Namibia. *Biological Conservation*, 93(3), pp.381-391.

Okello, M.M., Buthmann, E., Mapinu, B. and Kahi, H.C., 2011. Community opinions on wildlife, resource use and livelihood competition in Kimana Group Ranch near Amboseli, Kenya. *The Open Conservation Biology Journal*, 5(1).

Omondi, P., Bitok, E. and Kagiri, J., 2004. Managing human–elephant conflicts: the Kenyan experience. *Pachyderm*, 36, pp.80-86.

Osborn, F.V. and Anstey, S., 2002. *Elephant/human conflict and community development around the Niassa Reserve, Mozambique*. WWF-SARPO Report. Internet http://www.elephantpepper.org/downloads/Niassa% 20ele% 20report.pdf.

Osborn, F.V. and Parker, G.E., 2002. Community-based methods to reduce crop loss to elephants: experiments in the communal lands of Zimbabwe. *Pachyderm*, 33(32), p.e38.

Osborn, F.V. and Rasmussen, L.E.L., 1995. Evidence for the effectiveness of an oleo-resin capsicum aerosol as a repellent against wild elephants in Zimbabwe. *Pachyderm*, 20, pp.55-64.

Osborn, F.V., 2002. Capsicum oleoresin as an elephant repellent: field trials in the communal lands of Zimbabwe. *The Journal of Wildlife Management*, pp.674-677.

Osipova, L., Okello, M.M., Njumbi, S.J., Ngene, S., Western, D., Hayward, M.W. and Balkenhol, N., 2018. Fencing solves human-wildlife conflict locally but shifts problems elsewhere: A case study using functional connectivity modelling of the African elephant. *Journal of Applied Ecology*, 55(6), pp.2673-2684.

Parker, G.E. and Osborn, F.V., 2006. Investigating the potential for chilli Capsicum spp. to reduce human-wildlife conflict in Zimbabwe. *Oryx*, 40(3), pp.343-346.

Ponnusamy, V., Chackrapani, P., Lim, T.W., Saaban, S. and Campos-Arceiz, A., 2016. Farmers' perceptions and attitudes towards government-constructed electric fences in Peninsular Malaysia. *Gajah*, 45, pp.4-11.

Pozo, R.A., Coulson, T., McCulloch, G., Stronza, A. and Songhurst, A., 2019. Chilli-briquettes modify the temporal behaviour of elephants, but not their numbers. *Oryx*, 53(1), pp.100-108.

Raihan Sarker, A.H.M. and Røskaft, E., 2010. Human–wildlife conflicts and management options in Bangladesh, with special reference to Asian elephants (Elephas maximus). *International Journal of Biodiversity Science, Ecosystem Services & Management*, 6(3-4), pp.164-175.

Ramesh, G., Mathi, S., Pulari, S.R. and Krishnamoorthy, V., 2017, September. *An automated vision-based method to detect elephants for mitigation of human-elephant conflicts*. In 2017 International conference on advances in computing, communications and informatics (ICACCI) (pp. 2284-2288). IEEE.

Ramkumar, K., Ramakrishnan, B. and Saravanamuthu, R., 2014. Crop damage by Asian Elephants Elephas maximus and effectiveness of mitigating measures in Coimbatore Forest Division, South India. *International Research Journal of Biological Sciences*, 3(8), pp.1-11.

Ranjeewa, A.D., Tharanga, Y.J.S., Sandanayake, G.H.N.A., Perera, B.V. and Fernando, P., 2015. Camera traps unveil enigmatic crop raiders in Udawalawe, Sri Lanka. *Gajah*, 42, pp.7-14.

Scheijen, C.P., Richards, S.A., Smit, J., Jones, T. and Nowak, K., 2019. Efficacy of beehive fences as barriers to African elephants: a case study in Tanzania. *Oryx*, 53(1), pp.92-99.

Scrizzi, A., Le Bel, S., La Grange, M., Mabika, C.T., Czudek, R. and Cornélis, D., 2018. Urban human-elephant conflict in Zimbabwe: a case study of the mitigation endeavour. *Pachyderm*, (59), pp.76-85.

Sitati, N.W. and Walpole, M.J., 2006. Assessing farm-based measures for mitigating human-elephant conflict in Transmara District, Kenya. *Oryx*, 40(3), pp.279-286.

Sitati, N.W., Walpole, M.J. and Leader-Williams, N., 2005. Factors affecting susceptibility of farms to crop raiding by African elephants: using a predictive model to mitigate conflict. *Journal of Applied Ecology*, 42(6), pp.1175-1182.

Sitati, N., Leader-Williams, N., Stephenson, P.J., 2007. Mitigating Human-elephant conflict: case studies from Africa and Asia. Fauna & Flora International. *Mitigating Human-Elephant Conflict: Case Studies from Africa and Asia*. Matt Walpole and Matthew Linkie (Eds). pp. 37-46.

Smith, R. and Kasiki, S., 2000. *A spatial analysis of human-elephant conflict in the Tsavo ecosystem*. IUCN African Elephant Specialist Group Report, Kenya.

Sugiyo, A., Santo, A., Marthy, W. and Amama, F., *Evaluating the intervention methods to reduce human-elephant conflict around Way Kambas National Park*. International Wildlife Symposium at Bandar Lampung, Indonesia.

Taylor, R.D., 1993. Elephant management in Nyaminyami District, Zimbabwe: turning a liability into an asset. *Pachyderm*, 17, pp.19-29.

Thapa, S., 2010. Effectiveness of crop protection methods against wildlife damage: a case study of two villages at Bardia National Park, Nepal. *Crop Protection*, 29(11), pp.1297-1304.

Thouless, C.R. and Sakwa, J., 1995. Shocking elephants: fences and crop raiders in Laikipia District, Kenya. *Biological Conservation*, 72(1), pp.99-107.

Thuppil, V. and Coss, R.G., 2012. Using threatening sounds as a conservation tool: evolutionary bases for managing human–elephant conflict in India. *Journal of International Wildlife Law & Policy*, 15(2), pp.167-185.

Thuppil, V. and Coss, R.G., 2016. Playback of felid growls mitigates crop-raiding by elephants Elephas maximus in southern India. *Oryx*, 50(2), pp.329-335.

Van Eden, M., Ellis, E. and Bruyere, B.L., 2016. The influence of human–elephant conflict on electric fence management and perception among different rural communities in Laikipia County, Kenya. *Human Dimensions of Wildlife*, 21(4), pp.283-296.

Vollrath, F. and Douglas-Hamilton, I., 2002. African bees to control African elephants. *Naturwissenschaften*, 89(11), pp.508-511.

Von Hagen, R.L., 2018. *An Evaluation of Deterrent Methods Utilized to Prevent Crop Raiding by African Elephants (Loxodonta africana) in the Kasigau Wildlife Corridor, Kenya.* In Partial Fulfillment of the Requirements for the Degree Master of Science, Western Kentucky University.

Wahed, M.A., Ullah, M.R. and Irfanullah, H.M., 2016. *Human-elephant conflict mitigation measures: Lessons from Bangladesh.* International Union for Conservation of Nature, Bangladesh Country Office, Dhaka.

Warner, M.Z., 2008. *Examining human-elephant conflict in southern Africa: causes and options for coexistence*. University of Pennsylvania in Partial Fulfillment of the Requirements for the Degree of Master of Environmental Studies.

Wiafe, E.D. and Sam, M.K., 2014. Evaluation of a low-tech method, pepper–grease, for combatting elephant crop-raiding activities in Kakum Conservation Area, Ghana. *Pachyderm*, 55, pp.38-42.

Wijayagunawardane, M.P., Short, R.V., Samarakone, T.S., Nishany, K.M., Harrington, H., Perera, B.V.P., Rassool, R. and Bittner, E.P., 2016. The use of audio playback

to deter crop-raiding Asian elephants. *Wildlife Society Bulletin*, 40(2), pp.375-379.

Zeppelzauer, M., Hensman, S. and Stoeger, A.S., 2015. Towards an automated acoustic detection system for free-ranging elephants. *Bioacoustics*, 24(1), pp.13-29.

Zhang, L. and Wang, N., 2003. An initial study on habitat conservation of Asian elephant (Elephas maximus), with a focus on human elephant conflict in Simao, China. *Biological Conservation*, 112(3), pp.453-459.

Zimmermann, A., Davies, T.E., Hazarika, N., Wilson, S., Chakrabarty, J., Hazarika, B. and Das, D., 2009. Community-based human-elephant conflict management in Assam. *Gajah*, 30, pp.34-40.

Appendix 2: References featured in Table 1

The intervention techniques that were cited to be effective at reducing elephant (*Loxodonta spp.* and *Elephas maximus*) crop raiding, as determined by quasi-experimental, direct observation, human perception surveys, and elephant movement path designs. These techniques were reported among 79 of 95 studies that tested the efficacy of elephant crop-raiding interventions. As numerous techniques could have been tested among any given study, the same study may appear across multiple intervention techniques.

Intervention Technique

Chili pepper approaches

[1]Pozo, R.A., Coulson, T., McCulloch, G., Stronza, A. and Songhurst, A., 2019. Chilli-briquettes modify the temporal behaviour of elephants, but not their numbers. *Oryx*, 53(1), pp.100-108.

[2]Noga, S.R., Kolawole, O.D., Thakadu, O. and Masunga, G., 2015. Small farmers' adoption behaviour: uptake of elephant crop-raiding deterrent innovations in the Okavango Delta, Botswana. *African Journal of Science, Technology, Innovation and Development,* 7(6), pp.408-419.

[3]Dakwa, K.B., Monney, K.A. and Attuquayefio, D., 2016. Raid range selection by elephants around Kakum Conservation Area: Implications for the identification of suitable mitigating measures. *International Journal of Biodiversity and Conservation,* 8(2), pp.21-31.

[4]Wiafe, E.D. and Sam, M.K., 2014. Evaluation of a low-tech method, pepper–grease, for combatting elephant crop-raiding activities in Kakum Conservation Area, Ghana. *Pachyderm*, 55, pp.38-42.

[5]Zimmermann, A., Davies, T.E., Hazarika, N., Wilson, S., Chakrabarty, J., Hazarika, B. and Das, D., 2009. Community-based human-elephant conflict management in Assam. *Gajah*, 30, pp.34-40.

[6]Baishya, H.K., Dey, S., Sarmah, A., Sharma, A., Gogoi, S., Aziz, T., Ghose, D. and Williams, A.C., 2012. Use of chilli fences to deter Asian elephants-a pilot study. *Gajah*, 36, pp.11-13.

[7]Chelliah, K., Kannan, G., Kundu, S., Abilash, N., Madhusudan, A., Baskaran, N. and Sukumar, R., 2010. Testing the efficacy of a chilli–tobacco rope fence as a deterrent against crop-raiding elephants. *Current Science*, pp.1239-1243.

[8]Govind, S.K. and Jayson, E.A., 2013. Efficiency of chilli powder (Capsicum sp.) to deter wild elephants from the crop fields in a tropical area. *Millennial Zoology*, 14, pp.21-24.

[9]Davies, T.E., Wilson, S., Hazarika, N., Chakrabarty, J., Das, D., Hodgson, D.J. and Zimmermann, A., 2011. Effectiveness of intervention methods against crop-raiding elephants. *Conservation Letters*, 4(5), pp.346-354.

[10]Von Hagen, R.L., 2018. *An Evaluation of Deterrent Methods Utilized to Prevent Crop Raiding by African Elephants (Loxodonta africana) in the Kasigau Wildlife Corridor, Kenya.* In Partial Fulfillment of the Requirements for the Degree Master of Science, Western Kentucky University.

[11]Graham, M.D. and Ochieng, T., 2008. Uptake and performance of farm-based measures for reducing crop raiding by elephants Loxodonta africana among smallholder farms in Laikipia District, Kenya. *Oryx*, 42(1), pp.76-82.

[12]Kiiru, W., J. Kioko., and P. Granli. 2006. Mitigating human-elephant conflict in the Amboseli ecosystem, Kenya. Summary testing of deterrents Year 1. AERP HEC Project report to US Fish and Wildlife Service.

[13]Sitati, N.W. and Walpole, M.J., 2006. Assessing farm-based measures for mitigating human-elephant conflict in Transmara District, Kenya. *Oryx*, 40(3), pp.279-286.

[14]Branco, P.S., Merkle, J.A., Pringle, R.M., King, L., Tindall, T., Stalmans, M., Long, R.A., 2019. An experimental test of community-based strategies for mitigating human–wildlife conflict around protected areas. *Conservation Letters*, *13*(1), p.e12679

[15]Chang'a, A., Souza de, N., Muya, J., Keyyu, J., Mwakatobe, A., Malugu, L., Ndossi, H.P., Konuche, J., Omondi, R., Mpinge, A. and Hahn, N., 2016. Scaling-up the use of chili fences for reducing human-elephant conflict across landscapes in Tanzania. *Tropical Conservation Science*, 9(2), pp.921-930.

[16]Karidozo, M., Ferrel, V.O. 2015. Community Based Conflict Mitigation Trials: Results of Field Tests of Chilli as an Elephant Deterrent. *Journal of Biodiversity of Endangered Species* 3.

[17]Nyirenda, V.R., Chansa, W.C., Myburgh, W.J. and Reilly, B.K., 2011. Wildlife crop depredation in the Luangwa Valley, eastern Zambia. *Journal of Ecology and the Natural Environment,* 3(15), pp.481-491.

[18]Parker, G.E. and Osborn, F.V., 2006. Investigating the potential for chilli Capsicum spp. to reduce human-wildlife conflict in Zimbabwe. *Oryx*, 40(3), pp.343-346.

[19]Osborn, F.V., 2002. Capsicum oleoresin as an elephant repellent: field trials in the communal lands of Zimbabwe. *The Journal of Wildlife Management*, pp.674-677.

[20]Le Bel, S., La Grange, M. and Drouet, N., 2015. Repelling elephants with a chilli pepper gas dispenser: field tests and practical use in Mozambique, Zambia and Zimbabwe from 2009 to 2013. *Pachyderm*, 56:87-96.

[21]Osborn, F.V. and Rasmussen, L.E.L., 1995. Evidence for the effectiveness of an oleo-resin capsicum aerosol as a repellent against wild elephants in Zimbabwe. *Pachyderm*, 20, pp.55-64.

[22]Osborn, F.V. and Parker, G.E., 2002. Community-based methods to reduce crop loss to elephants: experiments in the communal lands of Zimbabwe. *Pachyderm*, 33(32), p.e38.

Crop guarding

[23]Raihan Sarker, A.H.M. and Røskaft, E., 2010. Human–wildlife conflicts and management options in Bangladesh, with special reference to Asian elephants (Elephas maximus). *International Journal of Biodiversity Science, Ecosystem Services & Management*, 6(3-4), pp.164-175.

[24]Nath, N.K., Lahkar, B.P., Brahma, N., Dey, S., Das, J.P., Sarma, P.K. and Talukdar, B.K., 2009. An assessment of human-elephant conflict in Manas National Park, Assam, India. *Journal of Threatened Taxa*, pp.309-316.

[25]Jasmine, B., Ghose, D. and Das, S.K., 2015. An attitude assessment of human-elephant conflict in a critical wildlife corridor within the Terai Arc Landscape, India. *Journal of Threatened Taxa*, 7(2), pp.6843-4852.

[26]Baskaran, N. 1995. Crop raiding by Asian elephant in Nilgiri Biosphere Reserve, South India. In: A week with Elephants: Proceedings of the International Seminar on the Conservation of the Asian Elephant (June 1993) (Eds J. C. Daniel and H. S. Datye) Oxford University Press, Bombay.

[27]Hedges, S. and Gunaryadi, D., 2010. Reducing human–elephant conflict: do chillies help deter elephants from entering crop fields?. *Oryx*, 44(1), pp.139-146.

[28]Sugiyo, A., Santo, A., Marthy, W. and Amama, F., *Evaluating the intervention methods to reduce human-elephant conflict around Way Kambas National Park*. International Wildlife Symposium at Bandar Lampung, Indonesia.

[29]Sitati, N.W., Walpole, M.J. and Leader-Williams, N., 2005. Factors affecting susceptibility of farms to crop raiding by African elephants: using a predictive model to mitigate conflict. *Journal of Applied Ecology*, 42(6), pp.1175-1182.

[30]Sitati, N., Leader-Williams, N., Stephenson, P.J., 2007. Mitigating Human-elephant conflict: case studies from Africa and Asia. Fauna & Flora International. Mitigating Human-Elephant Conflict: Case Studies from Africa and Asia. Matt Walpole and Matthew Linkie (Eds). pp. 37-46.

[31]Asimopoulos, S., 2016. Human-wildlife conflict mitigation in Peninsular Malaysia: lessons learnt, current views and future directions. Second cycle, A2E. Uppsala: SLU, Dept. of Urban and Rural Development.

[32]Thapa, S., 2010. Effectiveness of crop protection methods against wildlife damage: a case study of two villages at Bardia National Park, Nepal. *Crop Protection*, 29(11), pp.1297-1304.

[33]Haturusinghe, H.S. and Weerakoon, D.K., 2012. Crop raiding behaviour of elephants in the Northwestern region of Sri Lanka. *Gajah*, p.26.

[34]Bandara, R. and Tisdell, Cl., 2002. Asian Elephants as Agricultural Pests: Damages, Economics of Control and Compensation in Sri Lanka. *Natural Resources Journal*. 42.

[35]Gunaryadi, D. and Sugiyo, S.H., 2017. Community-based human–elephant conflict mitigation: The value of an evidence-based approach in promoting the uptake of effective methods. *PloS one*, 12(5).

[36]Hsiao, S.S., Ross, C., Hill, C.M. and Wallace, G.E., 2013. Crop-raiding deterrents around Budongo Forest Reserve: an evaluation through farmer actions and perceptions. *Oryx*, 47(4), pp.569-577.

[37]Nyirenda, V.R., Chansa, W.C., Myburgh, W.J. and Reilly, B.K., 2011. Wildlife crop depredation in the Luangwa Valley, eastern Zambia. *Journal of Ecology and the Natural Environment*, 3(15), pp.481-491.

[38]Nyirenda, V.R., Myburgh, W.J. and Reilly, B.K., 2012. Predicting environmental factors influencing crop raiding by African elephants (Loxodonta africana) in the Luangwa Valley, eastern Zambia. *African Journal of Environmental Science and Technology*, 6(10), pp.391-400.

[39]Gross, E.M., Lahkar, B.P., Subedi, N., Nyirenda, V.R., Lichtenfeld, L.L., Jakoby, O., 2019. Does traditional and advanced guarding reduce crop losses due to wildlife? A comparative analysis from Africa and Asia. *Journal of Nature Conservation* 50, 125712.

[40]Guerbois, C., Chapanda, E. and Fritz, H., 2012. Combining multi-scale socio-ecological approaches to understand the susceptibility of subsistence farmers to elephant crop raiding on the edge of a protected area. *Journal of Applied Ecology*, 49(5), pp.1149-1158.

Electric fences

[41]Davies, T.E., Wilson, S., Hazarika, N., Chakrabarty, J., Das, D., Hodgson, D.J. and Zimmermann, A., 2011. Effectiveness of intervention methods against crop-raiding elephants. *Conservation Letters*, 4(5), pp.346-354.

[42]Jayson, E.A. and Christopher, G., 2008. Human-elephant Conflict in the Southern Western Ghats: a Case Study from the Peppara Wildlife Sanctuary, Kerala, India. *Indian Forester*, 134(10), pp.1309-1325.

[43]Baskaran, N. 1995. Crop raiding by Asian elephant in Nilgiri Biosphere Reserve, South India. In: A week with Elephants: Proceedings of the International Seminar on the Conservation of the Asian Elephant (June 1993) (Eds J. C. Daniel and H. S. Datye) Oxford University Press, Bombay.

[44]Ramkumar, K., Ramakrishnan, B. and Saravanamuthu, R., 2014. Crop damage by Asian Elephants Elephas maximus and effectiveness of mitigating measures in Coimbatore Forest Division, South India. *International Research Journal of Biological Sciences*, 3(8), pp.1-11.

[45]Nyhus, P.J. and Tilson, R., 2000. Crop-raiding elephants and conservation implications at Way Kambas National Park, Sumatra, Indonesia. *Oryx*, 34(4), pp.262-274.

[46]Okello, M.M., Buthmann, E., Mapinu, B. and Kahi, H.C., 2011. Community opinions on wildlife, resource use and livelihood competition in Kimana Group Ranch near Amboseli, Kenya. *The Open Conservation Biology Journal*, 5(1).

[47]Evans, L.A., 2015. Fencing the front line: the separation of elephants and cultivation with electrified fences (Doctoral dissertation, University of Cambridge).

[48]Kioko, J., Kiringe, J. and Omondi, P., 2006. Human-elephant conflict outlook in the Tsavo-Amboseli ecosystem, Kenya. *Pachyderm*, 41, pp.53-60.

[49]Omondi, P., Bitok, E. and Kagiri, J., 2004. Managing human–elephant conflicts: the Kenyan experience. *Pachyderm*, 36, pp.80-86.

[50]Ponnusamy, V., Chackrapani, P., Lim, T.W., Saaban, S. and Campos-Arceiz, A., 2016. Farmers' perceptions and attitudes towards government-constructed electric fences in Peninsular Malaysia. *Gajah*, 45, pp.4-11.

[51]Asimopoulos, S., 2016. Human-wildlife conflict mitigation in Peninsular Malaysia: lessons learnt, current views and future directions. Second cycle, A2E. Uppsala: SLU, Dept. of Urban and Rural Development.

[52]O'Connell-Rodwell, C.E., Rodwell, T., Rice, M. and Hart, L.A., 2000. Living with the modern conservation paradigm: can agricultural communities co-exist with elephants? A five-year case study in East Caprivi, Namibia. *Biological Conservation*, 93(3), pp.381-391.

[53]De Boer, F., Ntumi, C.P., 2001. Elephant crop damage and electric fence construction in the Maputo Elephant Reserve, Mozambique. *Pachyderm* 30, 57–64.

[54]Nyirenda, V.R., Myburgh, W.J. and Reilly, B.K., 2012. Predicting environmental factors influencing crop raiding by African elephants (Loxodonta africana) in the Luangwa Valley, eastern Zambia. *African Journal of Environmental Science and Technology*, 6(10), pp.391-400.

Active defense (noise, projectiles, shots, fire)

[55]Adjewodah, P., Murphy, A. and Mason, J., 2003. Mitigating elephant crop-raiding: the Red Volta valley experience, Ghana. IUCN report, https://www.iucn.org/sites/dev/files/import/downloads/hecrvvrep.pdf

[56]Nath, N.K., Lahkar, B.P., Brahma, N., Dey, S., Das, J.P., Sarma, P.K. and Talukdar, B.K., 2009. An assessment of human-elephant conflict in Manas National Park, Assam, India. Journal of Threatened Taxa, pp.309-316.

[57]Sitati, N., Leader-Williams, N., Stephenson, P.J., 2007. Mitigating Human-elephant conflict: case studies from Africa and Asia. Fauna & Flora International. Mitigating Human-Elephant Conflict: Case Studies from Africa and Asia. Matt Walpole and Matthew Linkie (Eds). pp. 37-46.

[58]Musyoki, C., 2014. Crop defense and coping strategies: Wildlife raids in Mahiga'B'village in Nyeri District, Kenya.

[59]Nyumba, T., 2008. Coping with Human-Elephant Conflict in Laikipia District, Kenya (Doctoral dissertation, University of Cambridge).

[60]Thapa, S., 2010. Effectiveness of crop protection methods against wildlife damage: a case study of two villages at Bardia National Park, Nepal. Crop Protection, 29(11), pp.1297-1304.

[61]Bandara, R. and Tisdell, Cl., 2002. Asian Elephants as Agricultural Pests: Damages, Economics of Control and Compensation in Sri Lanka. Natural Resources Journal. 42.

[62]Le Bel, S., Taylor, R., Lagrange, M., Ndoro, O., Barra, M. and Madzikanda, H., 2010. An easy-to-use capsicum delivery system for crop-raiding elephants in Zimbabwe: preliminary results of a field test in Hwange National Park. *Pachyderm*, 47: 80-89.

Beehive fences

[63]Ngama, S., Korte, L., Bindelle, J., Vermeulen, C. and Poulsen, J.R., 2016. How bees deter elephants: beehive trials with forest elephants (Loxodonta africana cyclotis) in Gabon. PLoS One, 11(5).

[64]Nair, R.P. and Jayson, E.A., 2016. Effectiveness of beehive fences to deter crop raiding elephants in Kerala, India. Int. Res. J. Nat. Appl. Sci, 3, pp.14-19.

[65]King, L.E., Douglas-Hamilton, I. and Vollrath, F., 2011. Beehive fences as effective deterrents for crop-raiding elephants: field trials in northern Kenya. African Journal of Ecology, 49(4), pp.431-439.

[66]King, L.E., Lawrence, A., Douglas-Hamilton, I. and Vollrath, F., 2009. Beehive fence deters crop-raiding elephants. African Journal of Ecology, 47(2), pp.131-137.

[67]King, L.E., Lala, F., Nzumu, H., Mwambingu, E. and Douglas-Hamilton, I., 2017. Beehive fences as a multidimensional conflict-mitigation tool for farmers coexisting with elephants. Conservation Biology, 31(4), pp.743-752.

[68]Vollrath, F. and Douglas-Hamilton, I., 2002. African bees to control African elephants. Naturwissenschaften, 89(11), pp.508-511.

[69]Branco, P.S., Merkle, J.A., Pringle, R.M., King, L., Tindall, T., Stalmans, M., Long, R.A., 2019. An experimental test of community-based strategies for mitigating human–wildlife conflict around protected areas. Conserv. Lett.

[70]Scheijen, C.P., Richards, S.A., Smit, J., Jones, T. and Nowak, K., 2019. Efficacy of beehive fences as barriers to African elephants: a case study in Tanzania. *Oryx*, 53(1), pp.92-99.

Trenches

[71]Jasmine, B., Ghose, D. and Das, S.K., 2015. An attitude assessment of human-elephant conflict in a critical wildlife corridor within the Terai Arc Landscape, India. *Journal of Threatened Taxa*, 7(2), pp.6843-4852.

[72]Ramkumar, K., Ramakrishnan, B. and Saravanamuthu, R., 2014. Crop damage by Asian Elephants Elephas maximus and effectiveness of mitigating measures in

Coimbatore Forest Division, South India. *International Research Journal of Biological Sciences,* 3(8), pp.1-11.

[73]Sugiyo, A., Santo, A., Marthy, W. and Amama, F., *Evaluating the intervention methods to reduce human-elephant conflict around Way Kambas National Park*. International Wildlife Symposium at Bandar Lampung, Indonesia.

[74]Nyhus, P.J. and Tilson, R., 2000. Crop-raiding elephants and conservation implications at Way Kambas National Park, Sumatra, Indonesia. *Oryx*, 34(4), pp.262-274.

Detection systems

[75]Graham, M.D., Adams, W.M. and Kahiro, G.N., 2012. Mobile phone communication in effective human elephant–conflict management in Laikipia County, Kenya. *Oryx*, 46(1), pp.137-144.

[76]Sitati, N.W. and Walpole, M.J., 2006. Assessing farm-based measures for mitigating human-elephant conflict in Transmara District, Kenya. *Oryx*, 40(3), pp.279-286.

[77]Ramesh, G., Mathi, S., Pulari, S.R. and Krishnamoorthy, V., 2017, September. An automated vision-based method to detect elephants for mitigation of human-elephant conflicts. In 2017 International conference on advances in computing, communications and informatics (ICACCI) (pp. 2284-2288). IEEE.

[78]Zeppelzauer, M., Hensman, S. and Stoeger, A.S., 2015. Towards an automated acoustic detection system for free-ranging elephants. *Bioacoustics*, 24(1), pp.13-29.

Smart cropping

[79]Adjewodah, P., Murphy, A. and Mason, J., 2003. Mitigating elephant crop-raiding: the Red Volta valley experience, Ghana. IUCN report, https://www.iucn.org/sites/dev/files/import/downloads/hecrvvrep.pdf

[80]Neupane, D., Johnson, R.L. and Risch, T.S., 2017. How do land-use practices affect human—elephant conflict in nepal?. *Wildlife Biology*, 2017(4).

[81]Gross, E.M., McRobb, R. and Gross, J., 2016. Cultivating alternative crops reduces crop losses due to African elephants. *Journal of Pest Science*, 89(2), pp.497-506.

Playbacks

[82]Thuppil, V. and Coss, R.G., 2016. Playback of felid growls mitigates crop-raiding by elephants Elephas maximus in southern India. *Oryx*, 50(2), pp.329-335.

[83]Wijayagunawardane, M.P., Short, R.V., Samarakone, T.S., Nishany, K.M., Harrington, H., Perera, B.V.P., Rassool, R. and Bittner, E.P., 2016. The use of audio playback to deter crop-raiding Asian elephants. *Wildlife Society Bulletin*, 40(2), pp.375-379.

Decreasing rubbish availability

[84]Scrizzi, A., Le Bel, S., La Grange, M., Mabika, C.T., Czudek, R. and Cornélis, D., 2018. Urban human-elephant conflict in Zimbabwe: a case study of the mitigation endeavour. *Pachyderm*, (59), pp.76-85.

Spotlights

[85]Zimmermann, A., Davies, T.E., Hazarika, N., Wilson, S., Chakrabarty, J., Hazarika, B. and Das, D., 2009. Community-based human-elephant conflict management in Assam. *Gajah*, 30, pp.34-40.

Natural corridors

[86]Kikoti, A.P., Griffin, C.R., Pamphil, L. 2010. Elephant use and conflict leads to Tanzania first wildlife conservation corridor *Pachyderm*, 48, pp. 57-66
